# Supplementary material for: Observation of metallic electronic structure in a single-atomic-layer oxide
Source: Nat Commun. 2021 Oct 26;12:6171. doi: 10.1038/s41467-021-26444-z (PMC8548526; doi:10.1038/s41467-021-26444-z)
Supplement: Supplementary file 1 — Supplementary Information [file 41467_2021_26444_MOESM1_ESM.pdf]

Supplementary Information: Observation of metallic electronic structure in a single-atomic-layer oxide

Byungmin Sohn*[1,2], Jeong Rae Kim*[1,2], Choong H. Kim[1,2], Sangmin Lee[3], Sungsoo Hahn[1,2], Younsik Kim[1,2], Soonsang Huh[1,2], Donghan Kim[1,2], Youngdo Kim[1,2], Wonshik Kyung[1,2], Minsoo Kim[1,2], Miyoung Kim[3], Tae Won Noh†[1,2], and Changyoung Kim‡[1,2]

[1]Center for Correlated Electron Systems, Institute for Basic Science, Seoul 08826, Korea
[2]Department of Physics and Astronomy, Seoul National University, Seoul 08826, Korea
[3]Department of Materials Science and Engineering and Research Institute of Advanced Materials, Seoul National University, Seoul 08826, Korea

*These authors contributed equally to this work.
†Electronic address: twnoh@snu.ac.kr
‡Electronic address: changyoung@snu.ac.kr

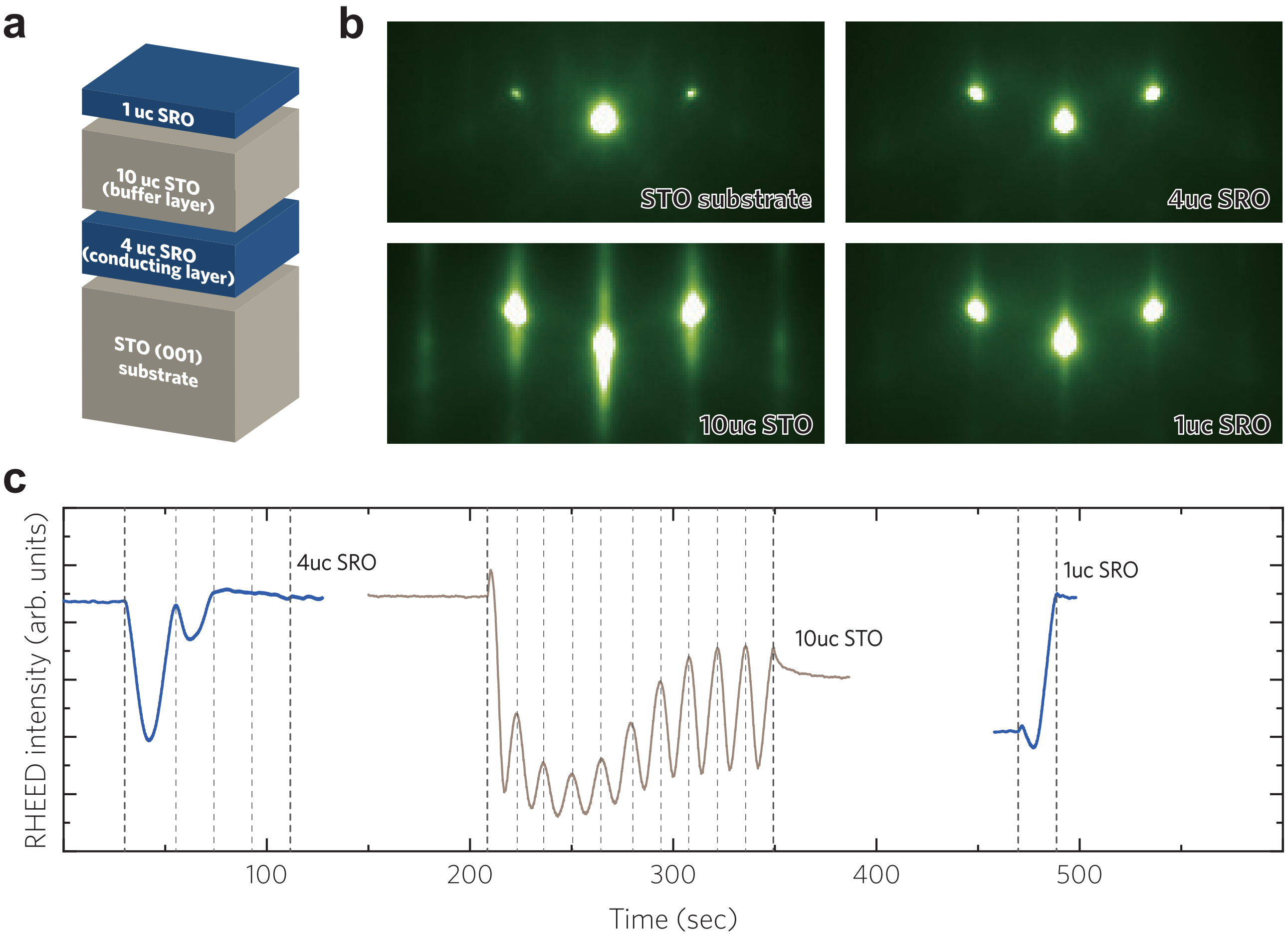

Supplementary Fig. 1: **Atomic-scale epitaxy of a charging-free ultrathin $SrRuO_3$ (SRO) heterostructure.** (**a**) A schematic of the charging-free ultrathin SRO heterostructure composed of 4 unit-cell (uc) SRO layer (conducting layer), 10 uc $SrTiO_3$ (STO) layer (buffer layer), and 1 uc ultrathin SRO layer, sequentially grown on a $SrTiO_3$ (001) substrate. (**b**) Reflection high-energy electron diffraction (RHEED) patterns along the $[100]_{cubic}$ direction captured during the growth of the layers. (**c**) In-situ monitoring of the RHEED intensity during the growth of the charging-free ultrathin SRO heterostructure.

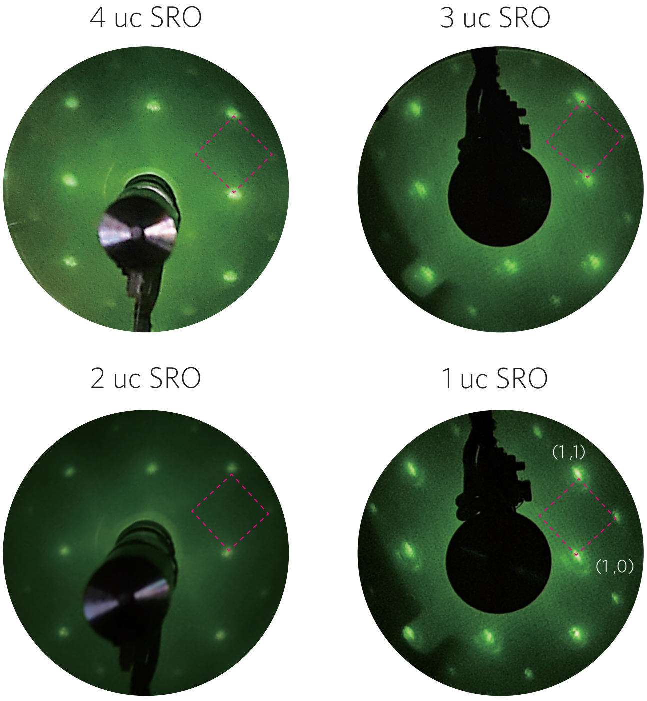

Supplementary Fig. 2: **Surface symmetry of ultrathin SRO layers characterized by low-energy electron diffraction (LEED).** 4, 3, 2, and 1 uc SRO films have $\sqrt{2} \times \sqrt{2}$ surface reconstruction. Pink dotted squares correspond to the surface primitive cell of SRO.

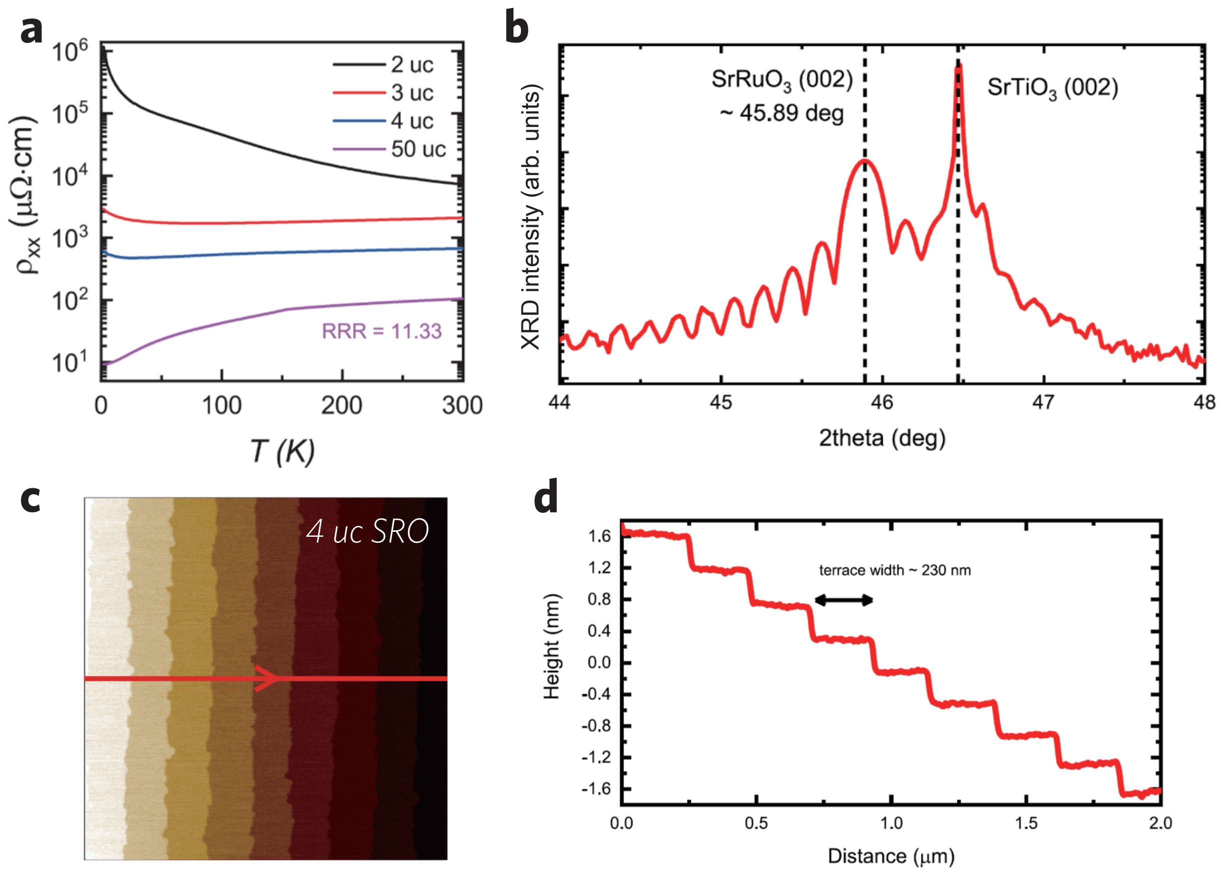

Supplementary Fig. 3: **Characterization of SRO thin films.** (**a**) Temperature dependent resistivity for 2, 3, 4 and 50 uc SRO thin films. The 50 uc SRO thin film exhibits a high residual resistivity ratio (RRR) value of 11.33 [reproduced from B. Sohn et al.[1]]. The 4 and 3 uc SRO films show metallic behavior at high temperature, whereas 2 uc sample shows only insulating behavior over the entire measured temperature range. We believe that extrinsic effects such as disconnected conducting path at step terraces[2] make it difficult to measure the intrinsic transport properties of ultrathin films. (**b**) X-ray diffraction 2θ-theta scan of 50 nm thick SRO film grown on STO (001) substrates. (**c, d**) An atomic force microscopy (AFM) image of a 4 uc SRO film. Step-terrace structure with 0.4 nm step height is present on the surface. The typical terrace width is around 230 nm.

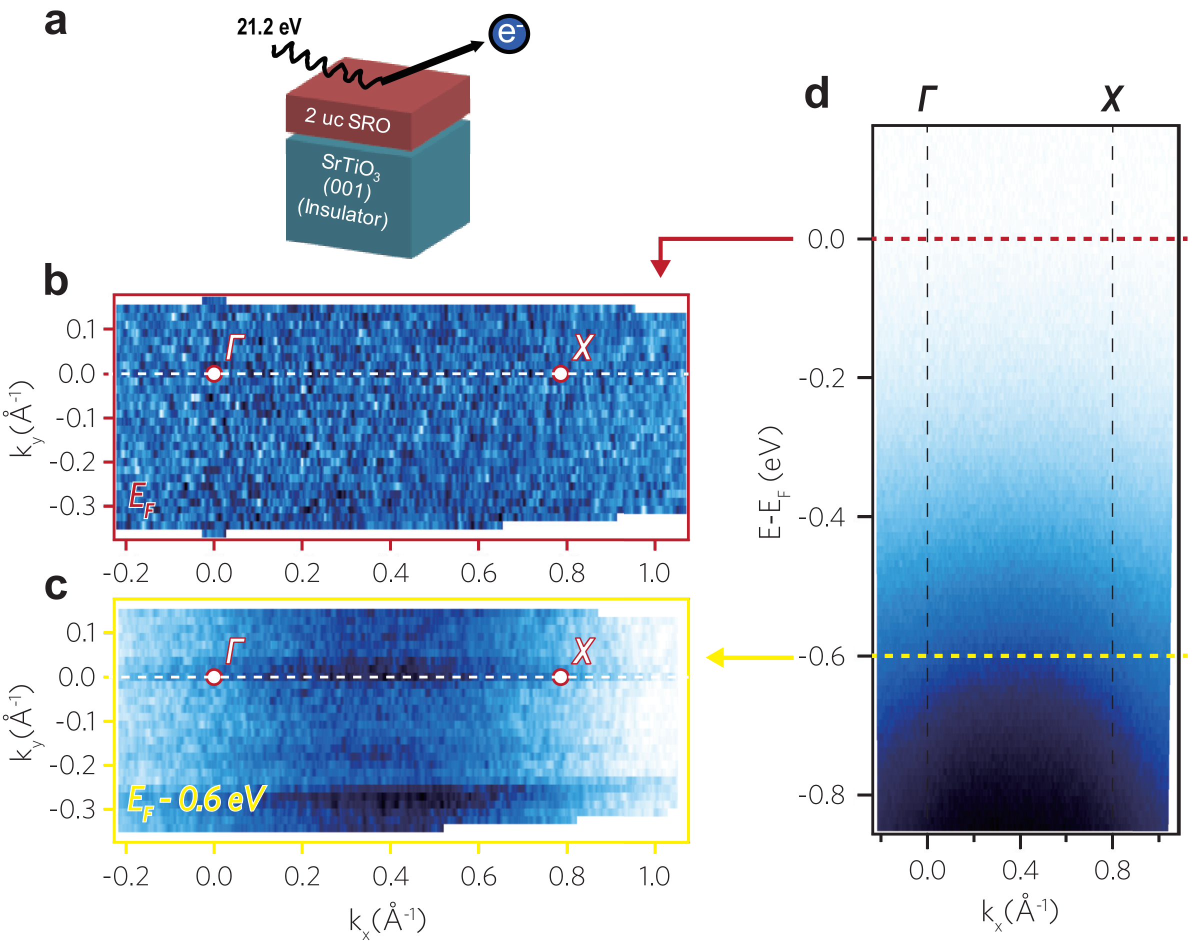

Supplementary Fig. 4: **Angle-resolved photoemission spectroscopy (ARPES) results of a 2 uc SRO thin film without a conducting layer.** (**a**) Schematic of 2 uc SRO on a STO (001) substrate. The system is subject to the charging effect without the conducting SRO layer. (**b, c**) Constant energy maps in the k_x-k_y plane at $E = E_F$ and $E_F - 600$ meV. (**d**) The Γ-M high-symmetry cut. No dispersive bands are observed in the constant maps and high-symmetry cut. We attribute the absence of bands to a charging effect. Therefore, a conducting layer (or a conducting substrate) is necessary to study thin films with ARPES.

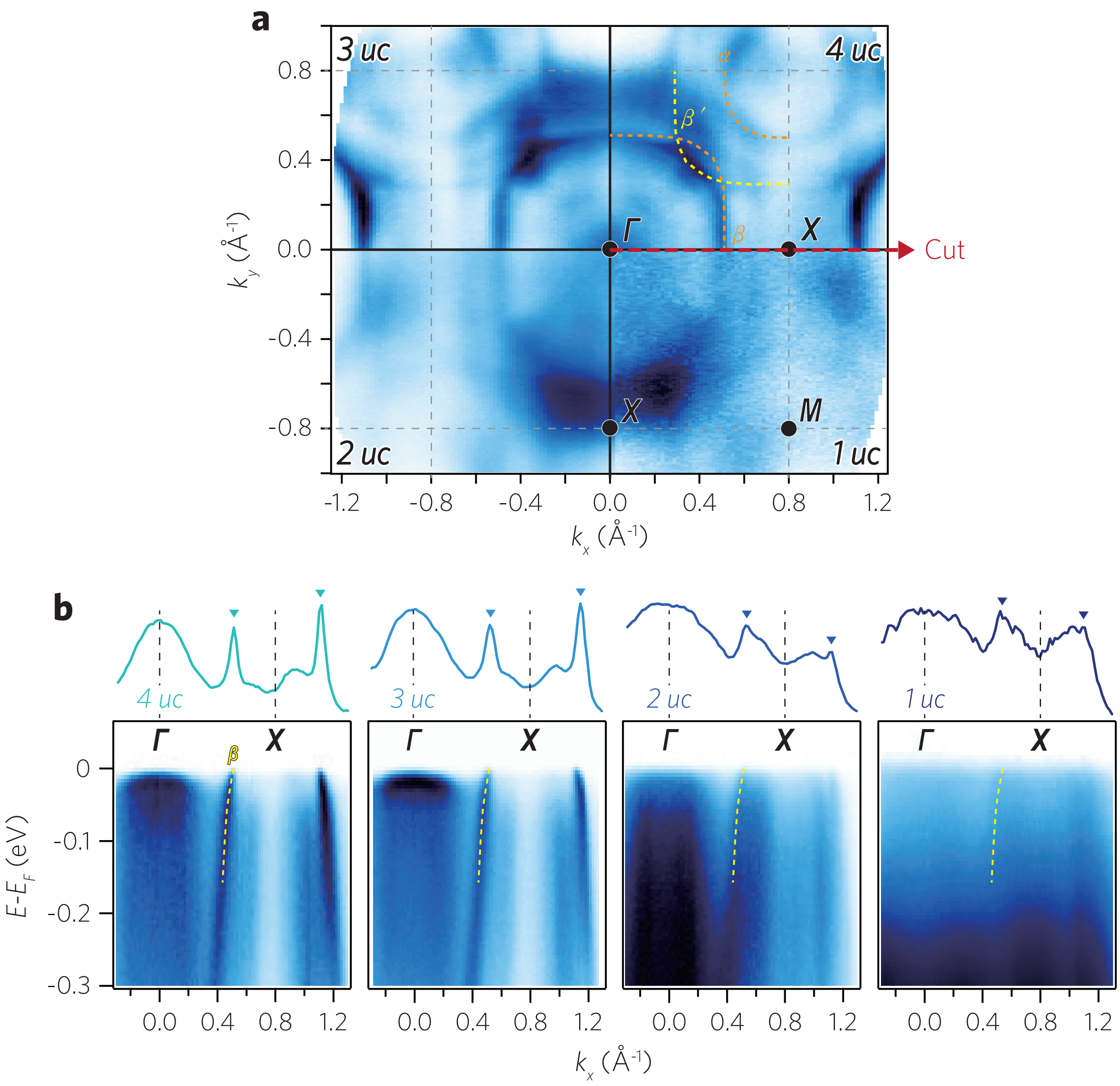

Supplementary Fig. 5: **Fermi surfaces (FSs) and band dispersions of SRO ultrathin films.** (**a**) FSs of ultrathin SRO layers with specified thicknesses (Fig. 2(**a**) in the main manuscript). (**b**) Γ-X high-symmetry cuts (Cut in (**a**)) and momentum distribution curves (MDCs) at E_F. The inverted triangles in the MDCs mark the peak for the β band which is indicated as a yellow dotted line. The β band is clearly resolved at all thicknesses.

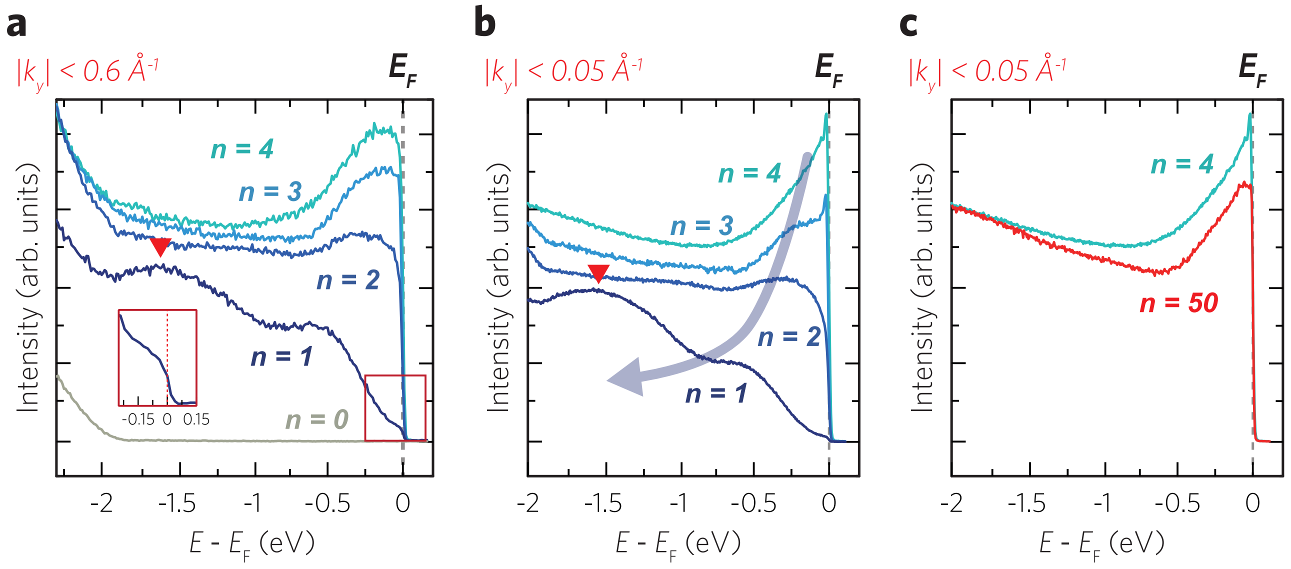

Supplementary Fig. 6: **Photoemission spectra from SRO heterostructures.** (**a**) Angle-integrated photoemission spectra integrated over the range of $|k_y| < 0.6$ Å^{-1} and $k_x = 0$ (Fig. 1(c) in the main manuscript). A red inverted triangle indicates a 'hump' feature. (**b**) Energy distribution curves (EDCs) of 4, 3, 2, and 1 uc SRO near the Γ point ($|k_y| < 0.05$ Å^{-1} and $k_x = 0$. A clear quasiparticle (QP) peak is observed for 4 and 3 uc SRO and spectral weight transfer of QP is observed with decreasing thickness. (**c**) EDCs of 50 and 4 uc SRO near the Γ point. The intensity of QP peak becomes weaker in 50 uc SRO thin film.

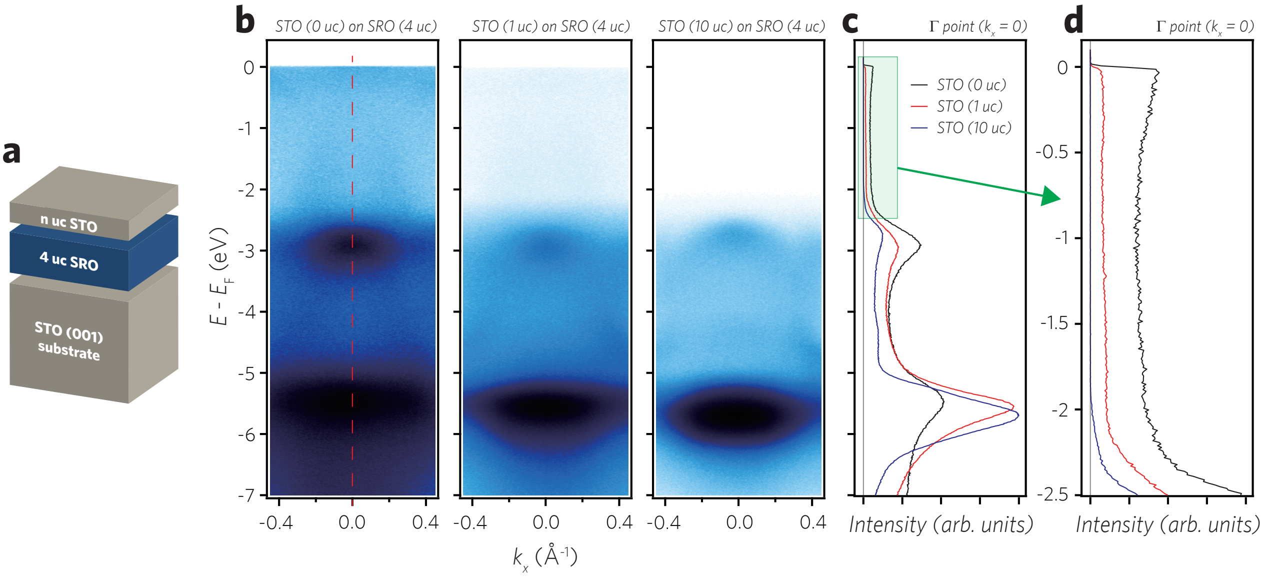

Supplementary Fig. 7: **Band dispersions of STO(n uc)/SRO(4 uc)/STO substrate.** (**a**) A schematic of STO(n uc)/SRO(4 uc)/STO (001) substrate heterostructure. (**b**) Γ-X high-symmetry cuts of 0, 1, and 10 uc STO grown on a 4 uc SRO. Spectral weight near the Fermi level decreases as the thickness of STO layers becomes thicker. (**c**) EDCs of 0, 1, and 10 uc STO grown on a 4 uc SRO near the Γ point (red dotted line in (**b**)). (**d**) Enlarged view of the spectra within the green rectangle in (**c**). Spectral intensity decreases as the STO layer becomes thicker and completely disappears for 10 uc STO layer.

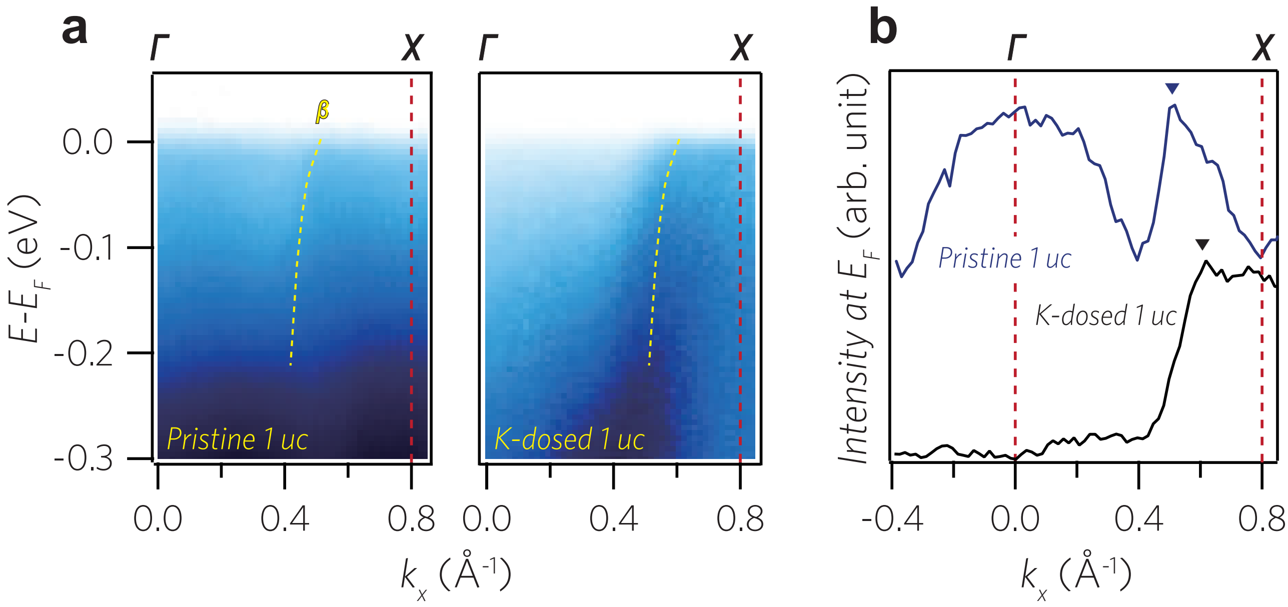

Supplementary Fig. 8: **Electron-doped monolayer SRO after K dosing.** (**a**) Γ-X high-symmetry cut of a 1 uc SRO (Cut in Supplementary Fig. 5(a)) before and after K dosing. A β band is indicated with a yellow dotted line. (**b**) MDCs at E_F. The inverted triangles represent the β band peak. After K dosing, the k_F of the β band changes from 0.52 to 0.60 $\AA^{-1}$.

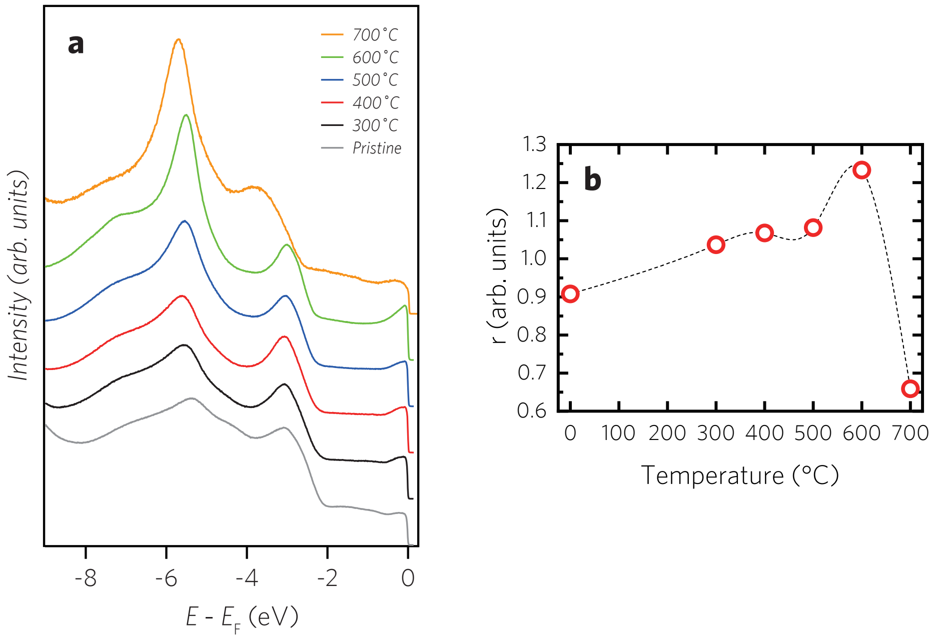

Supplementary Fig. 9: **Post-annealing-temperature-dependent photoemission spectra from 20 uc SRO thin films on a STO (001) substrate near the Γ point.** (**a**) Spectra before post-annealing (grey) and after post-annealing at 300 C° (black), 400 C° (red), 500 C° (blue), 600 C° (green), and 700 C° (orange). After annealing, a QP emerges. The intensity of QP increases with post-annealing temperatures but it eventually becomes weak after annealing at 700 C°. (**b**) The ratio of QP intensity in (a). r is defined as I_{QP}/I_{HB}, where I_{QP} is the intensity between E_F and $E_F - 0.1$ eV, and I_{HB} the integrated intensity between $E_F - 0.45$ eV and $E_F - 0.55$ eV. After post-annealing, r increases until 600 C° but decreases again when the SRO film is annealed at 700 C°.

Supplementary References

[1] Sohn, B. *et al.* Hump-like structure in hall signal from ultra-thin $SrRuO_3$ films without inhomogeneous anomalous Hall effect. *Curr. Appl. Phys.* **20**, 186–190 (2020).

[2] Lagally, M. G. & Zhang, Z. Thin-film cliffhanger. *Nature* **417**, 907–909 (2002).
